# Evaluation of P300 Response Through the Visual Oddball Paradigm in Healthy Children and Adolescents

**DOI:** 10.7759/cureus.89180

**Published:** 2025-07-31

**Authors:** Sangeeta Gupta, Arun Prasad

**Affiliations:** 1 Physiology, All India Institute of Medical Sciences, Gorakhpur, IND; 2 Paediatrics, All India Institute of Medical Sciences, Patna, IND

**Keywords:** adolescents, age, amplitude, children, event-related potential, latency, p300, visual

## Abstract

Background

P300 event-related potential (ERP) has been used to assess differences in modes of information processing and cognitive skill from childhood through adulthood. However, research on the development of the visual P300 is relatively sparse, with several discrepant findings. In this study, we aimed to evaluate the conventional parameters of visual ERPs and their relationship with mean response time in children and adolescents.

Methods

We conducted the study on 60 subjects in the age group of 5-18 years. P300 latency and N2-P3 amplitude were recorded by the visual oddball paradigm, and the age group comparisons were performed by one-way analysis of variance (ANOVA). Mean response time was calculated, and the correlation with P300 latency and amplitude was obtained by Pearson’s correlation coefficient (r). Statistical significance was set as p<0.05.

Results

We found a decrease in the mean P300 latency and an increase in N2-P3 amplitudes with age, with statistical significance (p<0.001) (one-way ANOVA). Changes were significantly pronounced until the ages of 8-11 years. P300 latency did not vary with significant correlation with the mean response time (r=0.1689, r²=0.029, p=0.19). N2-P3 amplitude showed a statistically significant moderate negative correlation with the mean response time (r=−0.59, r²=0.35, p<0.001) (Pearson correlation analysis).

Conclusion

In children and adolescents, age has a distinctive pattern of impact on visual P300 amplitude and latency. ERP data should be interpreted with careful consideration in children, and reference values should be used. Association of faster mean response time with larger amplitude found in the study raises the possibility that response speeds correspond to executive control efficiency.

## Introduction

In the field of cognitive neuroscience, event-related potentials (ERPs) are a vital research approach. ERPs consist of a series of positive and negative waves in response to a task-relevant stimulus. Researchers have described P3 or P300 as the third major positive peak, a late positive component, generated in response to infrequently attended, task-relevant stimuli of the auditory, visual, or somatic modalities [1,2]. Researchers have suggested that various specific psychological constructs, including stimulus discrimination, directed attention, sequential information processing, and short-term memory, are associated with P300 response [2,3].

The most prototypical experimental approach for cognitive and attention measurement in ERP research is the ‘oddball’ paradigm, which consists of a repetitive presentation of a standard stimulus with a randomly interspersed rare deviant stimulus. The deviant and standard stimuli differ in certain physical features. Subjects discriminate between two stimuli through behavioural tasks, such as pressing a button in response to deviants or silently counting the number of deviants (active paradigm), or they are instructed to perform another task (passive mode) [4]. In the auditory modality, the target or deviant stimuli differ from standard or non-target stimuli in one auditory attribute, e.g. frequency. In the visual modality, the target stimuli can differ in certain physical features (such as colour or shape). However, researchers have not evaluated the visual modality as extensively as they have evaluated auditory stimuli for ERP assessments.

As a neuroelectric measure of cognitive functions, the ERP has played a crucial role in quantifying and understanding age-related cognitive changes. The brain experiences a variety of regional structural architecture changes and intricate alterations in functional activation patterns from childhood to adulthood [5,6]. Polich (2007) reported that P300 can index the ability to allocate attention rapidly [7]. Because the P300 signal is linked to executive processes such as attentional control and inhibition, it is an excellent focus for research on the functional development of the brain throughout adolescence, when these cognitive abilities continue to develop significantly [8]. The oddball paradigm is the most widely employed experimental task, and age-related changes in P300 components have been the subject of several cross-sectional developmental studies [9]. According to extensive cross-sectional investigations on auditory P300 components and a meta-analysis, P300 amplitude rises with age [10,11]. However, the contrary has also occasionally been observed for P300 measured in visual paradigms, with amplitude decreasing with age [9,12]. Additionally, researchers have argued that the evolution of the P300 is modality-specific [6].

Visual ERPs have received relatively little attention in the literature compared to auditory ERPs, which have been the subject of numerous investigations. Consequently, visual ERP data have been sparse to compare the findings. Furthermore, previous researchers have seldom considered the effect of age on ERPs in childhood and adolescence in Indian subjects. In addition, researchers have rarely reported data on response time when responding to the oddball paradigm in children. Thus, the aim of this study is to evaluate the conventional parameters of visual ERPs and their relationship with mean response time in a cohort of Indian subjects in the age range from childhood to adulthood.

## Materials and methods

We studied 60 subjects in the age group of 5-18 years for a duration of 6 months (from August 2018 to February 2019). The study was conducted in the Neurophysiology Laboratory, Department of Physiology, All India Institute of Medical Sciences (AIIMS), Patna, Bihar, India. We recruited participants from outpatient clinics for ailments unrelated to neurological, psychiatric, or visual conditions. We confirmed normal neurological examination, and subjects with no signs of cognitive or behavioural impairment were included. All participants were right-handed (Edinburgh Handedness Inventory, Oldfield, 1971) and had normal or corrected-to-normal vision [13]. 

We obtained prior approval from the Institutional Ethics Committee (IEC Ref no. AIIMS/Pat/IEC/2018/281) for the study. We obtained consent forms that were appropriately filled out by the participants or their parents, as informed consent, before undertaking the tests. We categorised participants into four distinct cross-sectional samples that encompassed the following age categories in light of more pronounced age-related changes and recommendations for smaller age ranges in children and adolescents: 5-7, 8-11, 12-14, and 15-18 years [14]. We collected the data using a purposive sampling strategy, with 15 participants per age group. We matched subjects in terms of body temperature, grade point average, or academic performance, as well as the timing of food intake in relation to the test [15].

Procedure

**Figure 1:**
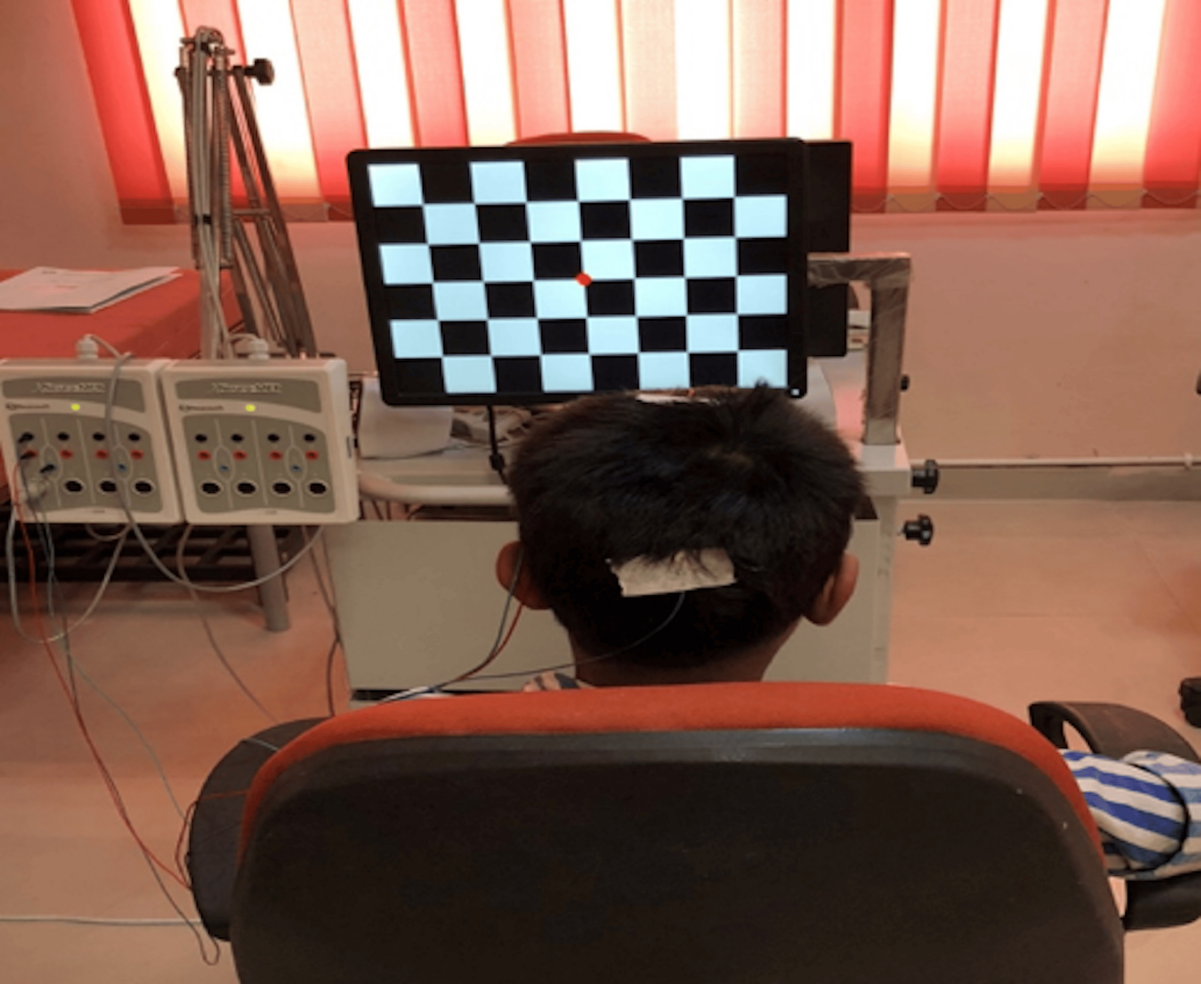
Recording setup of P300 response by visual oddball paradigm in a 15-year-old male participant

A visual P300 test was performed on Neuro-MEPω EMG and EP digital neurophysiological system software in the Neurophysiology Laboratory at AIIMS Patna. We performed a single-channel recording in a quiet environment. Appropriate skin preparation preceded surface electrode application (we employed the international 10-20 system). Gold-plated disc electrodes were applied to the forehead (Fpz) (ground) and the right mastoid (M2) (reference). Visual ERP waveforms were recorded from two active electrode locations (Cz and Pz) separately. The impedance of less than 5 kΩ (kiloohms) was ensured. We provided proper training and instructions to distinguish between the two types of stimuli (target and non-target or standard) before starting the test. The rare-frequent oddball paradigm consisted of blue-white and black-white checkerboard patterns (112 minutes, check size, reversal rate: 1 Hz) on a video monitor with a screen size of 26×33 cm (Figure 1). Thirty-five Hz high-cut and 0.5 Hz low-cut filters were used to filter the electrical signals. We employed a time window of 700 ms. We instructed subjects to press a button when they detected the target stimulus and not to respond when we presented the standard or non-target stimulus. 

Two records of 100 trials were recorded and analysed [16]. We excluded records with higher false positive errors (button press on a non-target stimulus) or false negative errors (no button press on a target stimulus) of ≥33% from the dataset. P300 latency was measured as the maximal positive peak after N2 within the range of 250 to 700 ms after stimulus presentation. P300 amplitude was measured from the peak of N2 to the peak of P300 (N2-P3). Mean reaction time (mean response time) was measured during the visual ERP test. All the responses recorded from Cz and Pz location were measured and analysed.

Statistical analysis

The data were organised in spreadsheets using Microsoft Excel software (Microsoft Corp., Redmond, WA, US). We compared P300 amplitude and latency among the four age groups using one-way ANOVA. For multiple comparisons across age ranges, we used the post hoc Tukey honestly significant difference (HSD) test. Pearson’s correlation coefficient (r) was calculated to find the relationship of P300 components with mean response time. Paired t-tests were used to compare the P300 latency and amplitude values recorded at Cz and Pz electrode sites. We considered p<0.05 as statistically significant. The analyses were performed using Stata statistical software version 12 (StataCorp LLC 4905 Lakeway Drive College Station, Texas 77845-4512 USA).

## Results

The mean age (± SD) of the participants was 11.9±4.24 years (age range: 5-18 years). Thirty participants (50%) were males and 30 (50%) were females. We compared P300 latency and N2-P3 amplitudes among the participants in four different age group categories (5-7 years (group 1), 8-11 years (group 2), 12-14 years (group 3), and 15-18 years (group 4); each with 15 participants; Table 1). 

Visual P300 latency and amplitude variation with age

P300 latency values of 335.53±17.44 ms at Cz and 341.53±14.9 ms at Pz (mean ± SD) in the childhood age group (5-7 years) decreased to 298.86±6.89 ms and 301.4±4.61 ms (at Cz and Pz, respectively) in the 15-18-year age group. The difference was statistically significant (p<0.001; one-way ANOVA; Table 1). We observed substantial latency reduction (up to 305.6±17.88 at Cz and 307.06±16.4 at Pz) in the 8-11 years age group (group 2). Although P300 latency decreased in later age groups, the decrease beyond that was not statistically significant for groups 2-3, 2-4, and 3-4 (p>0.05; Tukey HSD; one-way ANOVA; Table 1). 

N2-P3 amplitudes increased with age, with a statistically significant difference (p<0.001; one-way ANOVA; Table 1). Amplitudes (mean values in µv ± SD) increased from 3.57±0.36 (Cz) and 3.596±0.35 (Pz) in group 1 to 5.48±0.86 (Cz) and 5.49±0.84 (Pz) in group 4. Multiple comparisons with groups 2-3, 2-4, and 3-4 revealed a p-value >0.05, indicating that the considerable increase occurred until group 2 (8-11 years; Table 1).

**Table 1:** Visual P300 latency (ms) and N2-P3 amplitude (μV) in different age groups of the participants (5-18 years, n=60) *^1^p-value=9.18x10^-9^ (one-way ANOVA) *^2^p-value=7.83x10^-12^ (one-way ANOVA) *^3^p-value=0.000022 (one-way ANOVA) *^4^p-value=0.000012 (one-way ANOVA) The rare-frequent oddball paradigm consisted of blue-white and black-white checkerboards patterns (112 minutes, check size, reversal rate: 1 Hz) on a video monitor. n=15 for each age group SD: standard deviation; ms: milliseconds; µv: microvolts; ANOVA: analysis of variance; HSD: honestly significant difference; Hz: Hertz.

P300 component	Electrode location	Age-group (years)				F- value	p-value	Post hoc (Tukey HSD)
		5-7 (mean ± SD) group 1	8-11 (mean ± SD) group 2	12-14 (mean ± SD) group 3	15-18 (mean ± SD) group 4			
P300 latency (ms)	Cz	335.53±17.44	305.6±17.88	300.06±15.75	298.86±6.89	19.44	<0.001*^1^	1-2, 1-3, 1-4 (<0.001), 2-3, 2-4, 3-4 (p>0.05)
	Pz	341.53±14.9	307.06±16.4	302.73±14.17	301.4±4.61	30.55	<0.001*^2^	1-2, 1-3, 1-4 (<0.001), 2-3, 2-4, 3-4 (p>0.05)
N2-P3 amplitude (µv)	Cz	3.57±0.36	4.97±1.53	5.11±0.99	5.48±0.86	10.01	<0.001*^3^	1-2, 1-3, 1-4 (<0.001), 2-3, 2-4, 3-4 (p>0.05)
	Pz	3.596±0.35	4.99±1.43	5.12±0.99	5.49±0.84	10.70	<0.001*^4^	1-2, 1-3, 1-4 (<0.001), 2-3, 2-4, 3-4 (p>0.05)

**Figure 2:**
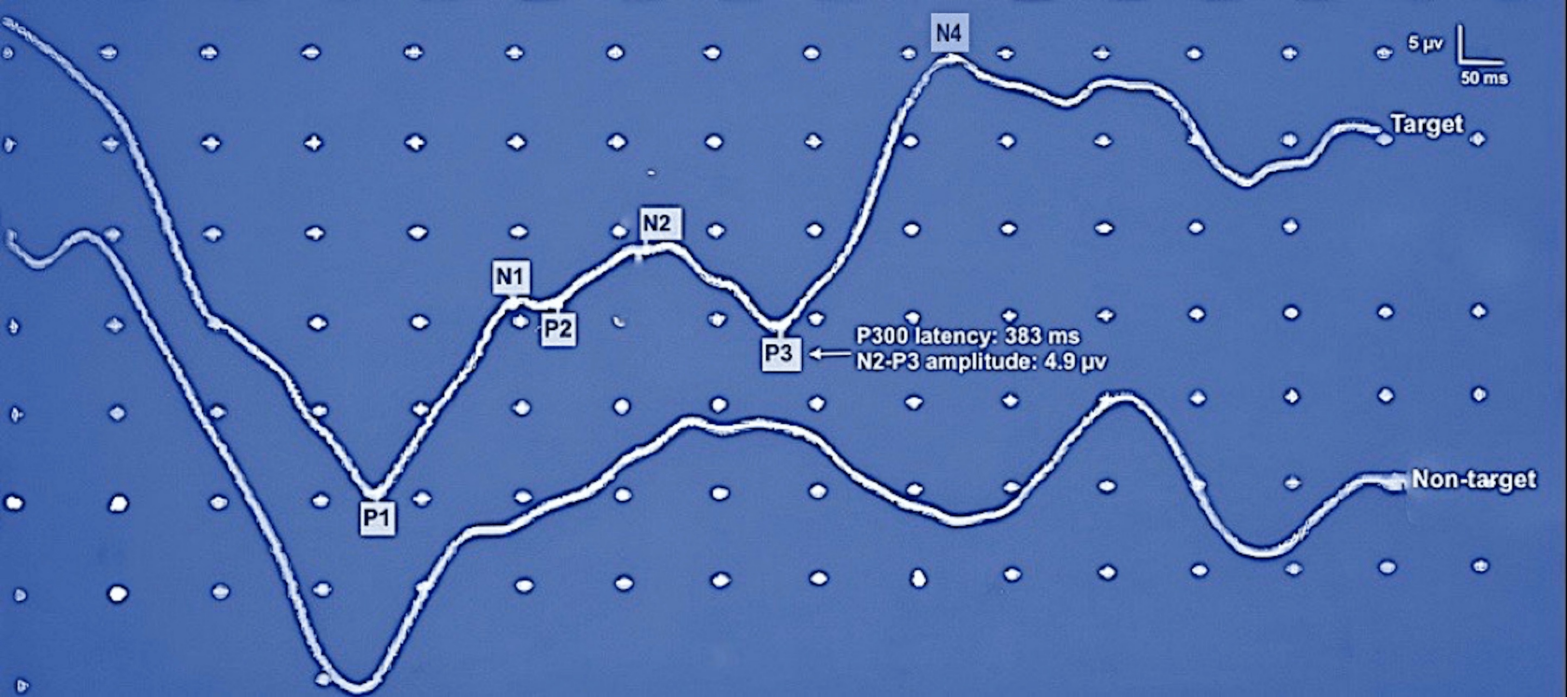
Visual P300 response of a 7-year-old male Representative visual P300 record of 5-7 years age group, recorded from midline parietal (Pz) location, showing increased P300 latency (383 ms) and relatively reduced N2-P3 amplitude (4.9 µv) in comparison with older age groups. The participant pressed the button as soon as he detected the rare/target stimuli within a series of the rare-frequent oddball paradigm consisting of blue-white and black-white checkerboards patterns (112 minutes, check size, reversal rate: 1 Hz). ms: milliseconds; µv: microvolts; Hz: Hertz.

**Figure 3:**
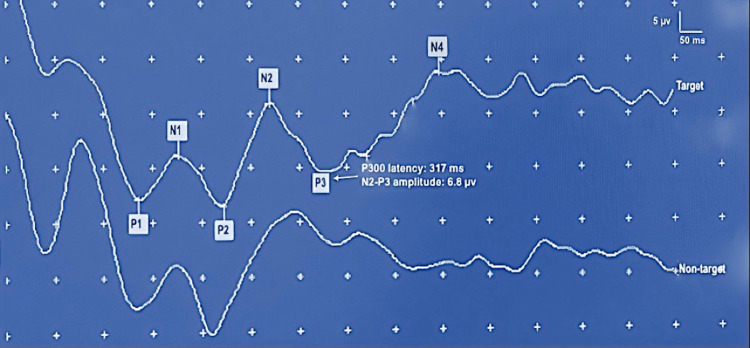
Visual P300 response of a 14-year-old male Representative visual P300 record of the 12-14 years age group, recorded from midline parietal (Pz) location, showing contrasting changes in P300 latency (317 ms) and N2-P3 amplitude (6.8 µv) in comparison with the 5-7 years age group. The participant pressed the button as soon as he detected the rare/target stimuli within a series of the rare-frequent oddball paradigm consisting of blue-white and black-white checkerboards patterns (112 minutes, check size, reversal rate: 1 Hz). ms: milliseconds, µv: microvolts, Hz: Hertz.

Figure 2 depicts a representative visual P300 record for group 1 (5-7 years), demonstrating increased P300 latency values (383 ms) while relatively lower N2-P3 amplitudes (4.9 µv). Figure 3 illustrates a record with a remarkably reduced latency value (317 ms) and amplitude augmentation (6.8 µv) in a participant from group 3 (12-14 years). We noted no significant variation in P300 latency values and N2-P3 amplitude with respect to electrode locations (Cz and Pz) in various age groups (p>0.05, paired t-test).

Visual P300 components and mean response time 

**Figure 4:**
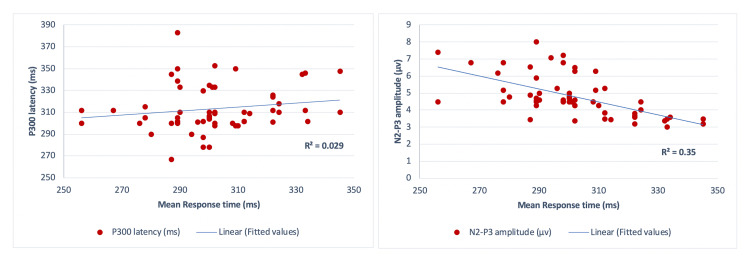
Scatter plot illustrating the relationship between P300 latency and mean response time (ms) (left) and between N2-P3 amplitude (µv) and mean response time (ms) (right), both at Pz No significant correlation observed between the P300 latency and mean response time (r=0.1689, r^2^=0.029, p=0.19), while significant negative correlation between N2-P3 amplitude and mean response time found (r=-0.59, r²=0.35, p<0.001) (Pearson correlation analysis) µv: microvolts; ms: milliseconds.

Mean response time (mean ± SD) of the study sample was 301.55±19.03 ms (Pz). We found no significant correlation of P300 latency with the mean response time (r=0.1689, r²=0.029, p=0.19). In contrast, N2-P3 amplitude varied with a significant negative correlation with the mean response time (Pz) (r=−0.59, r²=0.35, p<0.001) (Pearson correlation analysis, Figure 4).

## Discussion

We aimed to examine the relationship between age and visual P300 components in the early developmental ages, i.e., childhood and adolescence age groups. We also sought to examine the behaviour of P300 components with mean response time in the younger age groups. 

P300 latency demonstrated a characteristic decline in the childhood age group. We found that the majority of the latency reduction (up to 307.06±16.4 ms at Pz) was evident until the group 2 (8-11 years age group). It further decreased until the age of 18 years, but the difference was not statistically significant (groups 2-3, 2-4, and 3-4 (p>0.05); Tukey HSD; one-way ANOVA; Table 1). A greater decline until 11 years of age was therefore noticeable. Picton also suggested a more marked decrease in latency between the ages of 5 and 12 years, in line with our study [17]. Researchers have reported minimum latency values between 15 and 20 years of age [17]. We noted a similar decrease until 18 years of age. Katsanis et al. also reported a similar association between age and latency, indicating that P300 latency continues to decrease until late adolescence [16]. Tsai et al. found a similar association in children using the auditory oddball paradigm [18]. Previous researchers have reported a similar relation of P300 latency with age in children and adolescents [19-21]. However, Brown et al. reported no association between age and latency in a sample of 25 individuals who ranged in age from 15 to 45 years [22]. The above inconsistencies do not appear to be related to stimulus modality because researchers have found auditory and visual paradigms to have similar developmental trends during adulthood [23]. Variations in the methodologies used could explain the observed discrepancies. Researchers have described the P300 latency as a measure of the speed and efficiency of information processing in the brain. The plausible explanation offered for the decrease in latency observed during the adolescent years is the rapid development in the cortical association areas and the development of cognitive processes more effectively in terms of resource allocation with respect to time [10]. In addition, the visual P300 latency values obtained in our study (335.53±17.44 at Cz and 341.53±14.9 at Pz) were shorter than those of auditory P300s in young children [24].

We found the N2-P3 amplitude to be signiﬁcantly increased with age in our study. Notwithstanding, the major rise in amplitude values occurred in group 2 (8-11 years), and further changes were not statistically significant (p>0.05 for within-group comparisons: 2-3, 2-4, and 3-4). The findings align with previous studies [18,20]. It has been proposed that the P300 amplitude represents cognitive resources or brainpower that increases with age. Researchers have attributed the increase in P300 amplitudes to the rapid growth in information processing throughout early childhood [10]. The results indicate that the P300 components become stronger with age during development.

However, Katsanis et al. stated that the peak amplitudes of visual P300 diminished with increasing age among the participants in the 11-21 year age group [16]. We explain the above findings on the basis that the major rise in amplitude in our study (consistent with other similar studies) occurred in the age group of 8-11 years, and the mild increase further was not statistically significant. In contrast, the age range of the participants started with 11 years in the Katsanis study (Table 1). However, a few researchers on adolescents have indicated a negative age association in children and adolescents using the visual oddball paradigm [25,26]. This, in turn, lends the possibility of a different developmental trajectory of the P300 for auditory and visual modalities [6].

We did not observe an increase in P300 latency and amplitude at Pz (posterior scalp areas) in comparison with Cz (central scalp location) as reported in some previous studies (both P300 latency and N2-P3 amplitude increased at Pz site but with no statistical significance) [27].

Previous researchers have investigated the association of P300 and the decision process that invokes the response. In this regard, there is substantial evidence to support that the decision leading to a button press precedes the process that generates the P300 wave, and the subject can correctly press the button before the P300 peak [17]. In agreement with the above findings, the mean response time obtained in our research was about 13 ms earlier (301.55±19.03 ms) (Pz) than the mean P300 latency for the total study sample (313.18±21.12 ms) (at Pz). Kutas et al., in a study examining the relationship of P300 latency and response time, stated that P300 might mark the completion of stimulus evaluation [28]. 

When assessing the relationship of P300 latency and amplitude with mean response time in the present study, we obtained a moderate negative correlation of N2-P3 amplitude (p<0.001) with no significant correlation with respect to P300 latency (Pearson correlation analysis, Figure 4). Researchers have also found P300 latency to be unrelated to response time in previous reports [29]. The time required for the impulses to travel from the brain out to the muscles renders the possibility of P300 representing the decision process less likely [17]. A significant negative correlation between P300 amplitude and reaction time, as observed in a study by Holm et al., reveals additional aspects of the above association [30]. In another study measuring intra-individual reaction time variability, researchers found that the amplitude rather than the latency of the P300 was associated with faster and slower response times [31]. This result supports the hypothesis that variability in P300 amplitudes with the corresponding response speeds represents the efficiency of executive control [31].

Limitations

We did not include the age group of young adults in our study. This might have provided further insights into the age range where the visual P300 latency and amplitudes exhibited minimum and maximum values due to age-related variations. We did not record a visual P300 response from the Fz electrode location and, hence, could not consider frontal P300 responses for analysis. We did not seek gender effects in our study. 

## Conclusions

Visual ERP in children and adolescents demonstrates consistent and significant age-related changes. These age-related alterations in P300 amplitude and latency in children and adolescents are likely due to maturational changes in mental processing. An increase in amplitudes reflects an increase in neural power or cognitive resources that develop with age. The decline noted in P300 latency with age in younger individuals supports an increase in resource allocation efficiency in relation to time, owing to the ongoing cognitive development process. Considering the wide age-related variation in P300 latencies and amplitudes in children, ERP data should be interpreted with caution in children of various ages.

The association of a faster mean response time with a larger amplitude raises the possibility that response speeds correlate with the efficiency of executive control. Although the P300 response using the visual oddball paradigm demonstrates relatively similar age-related trends to those of auditory P300 responses, future studies would help to test whether P300 development differs between modalities by directly comparing age-related differences in P300 components using similar paradigms among the same participants.
